# Supplementary material for: Validation of the Infant and Young Child Development (IYCD) Indicators in Three Countries: Brazil, Malawi and Pakistan
Source: Int J Environ Res Public Health. 2021 Jun 6;18(11):6117. doi: 10.3390/ijerph18116117 (PMC8201322; doi:10.3390/ijerph18116117)
Supplement: Supplementary file 1 [file ijerph-18-06117-s001.zip › ijerph-1197332-supplementary.pdf]

File S1. Table demonstrating sampling framework for each country.

Age in months	Urban		Rural/Smaller Urban/Peri-urban (depending on setting)		
	Male	Female	Male	Female	Total
0–3	3	3	3	3	12
3–6	3	3	3	3	12
6–9	3	3	3	3	12
9–12	3	3	3	3	12
12–18	3	3	3	3	12
18–24	3	3	3	3	12
24–36	3	3	3	3	12
36–42	3	3	3	3	12
Total	24	24	24	24	96

File S2. Final IYCD tool with 100 items.

WHO Indicators of Infant and Young Child Development (IYCD)

Item guide

Notes:

- When the item or item description includes "few seconds" it refers to at least 3 seconds unless otherwise specified. If the caregiver asks for more clarifications, the interviewer can say "as long as counting 1,2,3".
- When the item or item description includes "several steps" it refers to at least 3–5 steps unless otherwise specified.
- Items that may need minor adaptation (of examples and objects) to ensure local relevance are flagged with an asterisk (*). Any adaptation must undergo approval from the IYCD team.

Item code	Item question	Description
**FINE AND GROSS MOTOR**		
MOT1	Does your child try to move his/her head (or eyes) to follow an object or person?	The child appears interested in objects and persons that he/she sees. When a person walks by in front of child, he/she tries to move his/her head and/or eyes to follow the object or the person.
MOT2	While your child is on his/her back, does he/she bring his/her hands together?	While lying down on his/her back, the child is able to bring his/her hands together in the center of the body over his/her chest.
MOT3	Does your child put objects or hands to his/her mouth?	The child is able to intentionally put either an object or his/her own hands to his/her mouth. The item is passed even if the movement is not completely controlled.

Item code	Item question	Description
MOT4	Does your child hold his/her head steady for at least a few seconds, without it flopping to the side?	While being held upright in the caregiver's arms or in a sitting position, the child is able to control his/her head for a few seconds (at least 3), without it flopping to the side and without the caregiver's hand supporting it.
MOT5	When he/she is on his/her tummy, does your child hold his/her head straight up, looking around for more than a few seconds?<br><br>*He/she can rest on his/her arms while doing this.*	When the child is lying on his/her tummy, he/she is able to lift his/her head and hold it straight up, looking around, for more than a few seconds (at least 3). He/she can elevate the chest with the arms.
MOT6	Does your child try to reach for objects that are in front of him/her by extending one or both arms?	While in sitting position, the child is able to reach for the object (toys, food, household objects) that are in front of him/her by extending one or both arms. Even if he/she does not grab the object, he/she is able to reach for it.
MOT7	Does your child reach for AND HOLD an object, at least for a few seconds?	The child is able to reach for the object and lift it for 3 seconds or more.
MOT8	Does your child sit WITH support, either leaning against something (furniture or person), or by leaning forward on his/ her hands?	The child is able to sit for 30 seconds, WITH support (either leaning against something, furniture or person, or by leaning forward on his/her hands). He/she may lose balance once or twice but is able to correct him/herself (with your slight support).
MOT9	If an object falls to the grounds, does your child look for it?	The child is able to look for an object of interest to him/her (by moving his/her eyes, head and/or body) that falls to the ground or that is taken away from him/her.
MOT10	Does your child drink from an OPEN cup while you hold it?	The child is able to, or attempts to, hold a cup without a lid, without spilling the liquid, when it is offered to him/her by holding it or putting his/her hand on the hand of the person supporting it.
MOT11	Does your child bang objects together, or bang an object on the table or on the ground?	The child is able to purposefully bang objects together (e.g., a spoon on a cup or plate), or to purposely bang on a table or a floor surface.
MOT12	Does your child pass an object from one hand to the other?	The child is able to pick up toys or pieces of food that are in front of him/her: he/she picks up one and then transfers it to the other hand without dropping it and then picks up the other one.
MO13	Does your child sit upright, with fairly straight back and neck control, without holding on to you, an object, or resting hands on the floor?	The child is able to sit upright for 30 seconds, with fairly straight back and neck control, without the help of a person, an object for support, or resting hands on the floor.
MOT14	Does your child pick up small items, like pieces of food USING THUMB AND FINGER TIPS?	The child is able to pick up small items, such as food or pieces of food, small stones or rocks, using thumb and pads or tips of one or more small fingers (NOT by using the palm), without dropping the item for at least 3 seconds.

Item code	Item question	Description
MOT15*	Does your child intentionally SQUEEZE or PAT/HIT an object that makes a noise in order to hear it?<br><br>*For example, squeezing or hitting an empty plastic bottle, or other object, to make noise? Not shaking or banging, but deliberately patting or squeezing it?*	The child is able to intentionally make noise with a squeeze toy (or something similar) by deliberately patting or squeezing it.<br>(Note: the example of the object used should be adapted for local relevance.)
MOT16	Does your child pull him/herself up from the floor while holding onto something for support?<br><br>*For example, can he/she pull him/herself up using a chair, a person, or some other object?*	The child is able to pull him/herself to a standing position from a seated position using a chair, table leg or other convenient object for support.
MOT17	Does your child pick up small items, like pieces of food using thumb and JUST ONE FINGER TIP?	The child is able to pick up small items (e.g., piece of maize, bead or small piece of cereal, etc.) in front of him/her with just the thumb and one fingertip, without dropping it for at least 3 seconds.
MOT18	When someone holds one or both hands (just to balance), or when your child is holding furniture or other object WITH JUST ONE HAND, does he/she make a few steps forward, without tripping?	When someone holds one or both hands (just to balance), or when the child is holding furniture or other objects WITH JUST ONE HAND, he/she is able to make at least three steps without tripping.
MOT19	Can your child push a toy car intentionally, all four wheels on the ground?	The child is able to slowly push the car intentionally. He/she makes it go, moving it back and forth.<br>(Note: If caregiver says that toy car is not available to the child, record as "don't know" and write "not applicable" in the notes.)
MOT20	Does your child stand up WITHOUT holding onto anything, even if just for a few seconds?	When in a standing position, the child is able to stand for a few seconds (at least 3) without any support.
MOT21	Does your child make any marks on paper or on the ground with a crayon/ pencil/ pen or a stick?	The child is able to make marks on paper or on the ground with a crayon/pencil/pen or a stick, such as to scribble, draw a picture or write. He/she is able to make intentional marks, in any manner. These can be light marks but should not be stabs.
MOT22*	Does your child climb onto an object (rock, porch, step, chair, bed, low table etc.)?	The child is able to pull him/herself without support onto a chair, bed, low table or other objects, for example, to reach something or to get onto a porch or a stoop.<br>(Note: The example of the object should be adapted for local relevance.)
MOT23	Does your child bend down or squat to pick up an object from the floor and then stand up again, without help from a person or object?	The child is able to bend over at the waist and/or knees or squat to pick up an object from the floor and then stand up again, without help from a person or object.
MOT24	Does your child take several steps (3–5) forward without holding onto any person or object for support, even if he/she falls down immediately?	The child is able to take 3 or more steps forward without holding onto any person or object for support, even if even if clumsy or stiff-legged or if he/she falls down immediately.

Item code	Item question	Description
MOT25	Does your child make a scribble on paper, or on the ground, in a BACK AND FORTH manner?<br><br>*For example, can he/she move the crayon/ pencil/ pen or stick back and forth?*	The child is able to make intentional marks on the paper or on the ground by scribbling back and forth.
MOT26	Does your child drink WITHOUT HELP from an OPEN cup?	The child is able to drink from an open cup on his/her own without any help and without spilling the liquid.
MOT27	Does your child walk well, with coordination without falling down often?<br><br>*With one foot in front of the other (rather than shifting weight side to side, stifflegged)?*	The child is able to walk well, with coordination and steady balance, with one foot in front of the other (rather than shifting weight side to side, or not bending his/her knees).
MOT28*	While standing, does your child purposefully THROW the ball and not just drop it?	The child is able to purposefully throw the ball, extending his/her arm using underhand or overhand motion and not just dropping the ball.<br>(Note: The item intends to measure the swinging movement of the arm with the release of the object to distance. If balls may not be available in the context, the example of an additional object should be added for local relevance.)
MOT29	Does your child unscrew a lid, at least turning the lid one rotation?	The child is able to take the lid off a jar by making at least one turning rotation.
MOT30	Does your child stack at least two objects on top of each other, such as bottle tops, blocks, stones, etc.?	Using at least two objects, the child is able to "make a tower" that does not immediately fall over.
MOT31	While standing, does your child kick a ball by swinging his/her leg forward?	The child is able to kick a ball, while standing, by swinging his/her leg back and then forward to make contact with the ball and to push it forward.
MOT32	Does your child run well, without falling or bumping into objects?	The child is able to run more than a few steps, with his/her feet lifted up and only one foot on the ground at any given time, without falling or bumping into objects.
MOT33	Does your child stand on one foot WITH SUPPORT by holding onto something or somebody?	The child is able to lift his/her foot and stand on one foot alone for 1 or more seconds without any support (either from a person or by holding onto an object or wall).
MOT34	Does your child walk sideways, two or more steps WITHOUT any support?	The child is able to walk sideways for two or more steps without any support (either from a person or by holding onto an object or wall).
MOT35	Does your child run and kick a ball, and do so successfully?	The child is able to successfully run up to and kick a ball as in playing football. He/she is able to swing the leg back and to balance on one leg to complete the kick and push the ball forward.<br>(Note: Success is measured by the child swinging the leg and moving the ball forward without falling.)

Item code	Item question	Description
MOT36	Does your child jump WITH BOTH FEET LEAVING THE GROUND AT ABOUT THE SAME TIME?	The child is able to jump, even if slightly, so that both feet leave the ground more or less together (not hopping on one foot).
MOT37	If you draw a straight line does your child do it, just as you did?	The child is able to draw a straight line on paper or in the ground with a crayon/ pencil/pen or a stick in any direction
MOT38	If you draw a circle does your child do it, just as you did?	The child is able to draw a circle on paper or in the ground with a crayon/ pencil/pen or a stick.
MOT39	Does your child stand on one foot WITH-OUT any support for at least a few sec-onds?	The child is able to stand on one foot WITHOUT any support (either from a person or by holding onto an object or wall) for a few seconds (at least 3).
MOT40	Does your child dress him/herself com-pletely (except for shoelaces, buttons and zippers)?	The child is able to put on his/her own clothes. As a result of this action the child is fully dressed (ex-cept for shoelaces, buttons and zippers) without any help.<br>(Note: Fully is determined by what is appropriate for the local context.)
**LANGUAGE AND COGNITIVE**		
LAN1	Does your child turn his/her head towards your voice or some noise?	The child is able to turn his/her head toward your voice or some noise (e.g., a bell or a rattle).
LAN2	When you TALK to your child, does he/she smile, make noises, or move arms, legs or trunk in response?	When someone talks to the child, he/she is able to smile, make noises (laughs, coos or makes other vocalizations), or move arms, legs or trunk in re-sponse.
LAN3*	Does your child make sounds when LOOKING at toys or people (not crying)?	The child is able to make sounds (squealing noises, high pitched sounds or grunts, groans, throaty sounds) when LOOKING at toys or people. This does not include crying as a response.<br>(Note: The example of the sounds should be adapted for local relevance.)
LAN4	Does your child make single sounds like "buh" or "duh" or "muh"?	The child is able to make 2 distinct consonant sounds as opposed to just vowels, including sounds such as "buh" or "duh" or "muh".
LAN5	Does your child make noise or gesture TO GET YOUR ATTENTION?	If your child wants your attention, the child is able to make noise or movement (e.g., moving, kicking, making noises, reaching out arms, etc.). The noise or movement may not necessarily be in the direc-tion of a person or object, but it communicates that the child wants something from the caregiver. For example, if your child is lying down and kicks his or her legs, flails arms, or makes noises to get a re-sponse from you.<br>[Note: The behavior is to get the caregiver's general attention, not for any other specific purpose.)

Item code	Item question	Description
LAN6*	Does your child make two similar sounds TOGETHER like baba, mumu, pepe (single consonant vowel combinations)?	The child is able to make consonant-vowel combinations such as “baba”, “mumu”, “pepe”.<br>(Note: The example of the sounds should be adapted for local relevance.)
LAN7	Does your child point, shake head, or otherwise gesture to let you know WHAT HE/SHE WANTs?	The child is able to let you know what he/she wants (e.g., wants something or wants to show you something or wants to be lifted or held), or is able to get your attention by specific gestures, such as pointing, shaking head, lifting arms to ask to be picked up. For example, the child can reach for his/her bottle (or other item) to indicate he/she wants it; he/she may shake head to indicate "no"; he/she may push away an object to indicate he/she does not want to play with it; he/she reaches to be picked up. Vocalizations may be present, but the gesture alone must be sufficient to indicate the child is communicating.
LAN8	Does your child say at least one word?	The child is able to spontaneously and appropriately say one meaningful word that applies to a specific object or situation.<br>(Note: A word is considered meaningful if it is consistently used and understood for a specific object in the home.)
LAN9	Does your child try to SHOW you something by reaching at you, pulling on your hand or clothes, using gestures, making noises (not words)?	The child is able to get your attention to show you something by performing any of the following actions: reaching at you, pulling on your hand or clothes, using gestures (points,), making noises and not words (grunts, moans). Your child is doing this to especially show you something specific, not just to get your attention or to indicate a need.
LAN10	Does your child put together noises that sound sentence-like, even if they are not words?	The child is able to make noises (even if not words) that sound sentence-like, to himself or in response to an adult making sounds. He/she jabbers expressively: he/she uses vocal inflections (alterations in pitch or tone) that are expressive and somewhat imitative of conversational inflections.
LAN11*	If you or someone else invites your child to play a familiar game (peek-a-boo, wave bye-bye, etc.), does your child know what to do without you showing them?<br><br>*Does he/she understand, even if you don't show him/her what to do? Does he/she try to participate, even if it's not exactly correct? Does he/she attempt the movement, activity, or response?*	The child is able to respond to someone asking him/her to play a game (peek-a-boo, bye-bye, etc.), even without you showing him/her what to do. He/she understands what is being asked and tries to participate. He/she attempts the movement, activity, or response, even if it's not exactly correct.<br>(Note: The examples of the game should be adapted for local relevance.)

Item code	Item question	Description
LAN12	Does your child identify at least one object? For example, when you ask "where is the ball/spoon/cup/cloth/door/plate/bucket etc." does your child look at or point to (or even name) the object?	The child is able to identify by looking or pointing at least one object commonly found around the house (e.g., cup, spoon, plate, pencil or pen, book, ball, soap, torch, candle, etc., to be determined).
LAN13	Without you making a gesture, does your child follow a simple spoken command or direction?<br><br>*Does the child understand when you ask something? Will he/she do at least one thing you ask the child to do? Can your child do it without you using a gesture?*	Without you making a gesture, the child is able to understand when you ask something and to follow a simple spoken command or direction (e.g., "come here", "sit down", "give it to me").<br><br>The child is able to understand a simple spoken command without the help of a gesture.
LAN14*	Does your child ask for something (e.g., food, water) by name when he/she wants it?	The child is able to use meaningful words to ask for something he/she desires (e.g., food, water, breastfeeding, being picked up, toy, go outside, etc.). Children of this age usually use single word utterances, spoken in a commanding tone and/or accompanied by a gesture. Words that the child may use include up, down, more, mine, no, want, please, bottle, etc.<br>(Note: The example of the words should be adapted for local relevance.)
LAN15*	Does your child usually understand what you say to him/her when you say it only once?<br><br>*For example, you don't have to repeat what you said to be understood.*	The child is able to understand what you say to him/her without having to repeat the message (e.g., when you ask the child to do something or tell him a simple story or sentence like "daddy is going now").<br>(Note: The example of the sentence should be adapted for local relevance.)
LAN16	Does your child identify at least one body part? For example, if you ask "where's your eyes?" can he/she point to his/her eyes?	The child is able to identify at least one body part (e.g., nose, eyes, ears, mouth, head, foot, teeth, arm, belly, etc.) when asked or when an adult points to them. Some children may gesture (blink, stick out tongue) rather than point to body part. This is acceptable.
LAN17	Does your child follow directions with more than one step?<br>*For example, "Go to the kitchen and bring me a spoon"?*	The child is able to follow complex directions with more than one step (e.g., get me your cup and put water in it) without making any gesture.
LAN18*	Does your child use two WORDS together in a meaningful phrase/speak in short two word sentences?<br><br>*For example, "mama go," "give mama," "daddy gone," etc.*	The child is able to make spontaneous two-word or multiple-word utterances in a meaningful phrase. Common examples of this type of utterance include "You do", "Where dada?" "Want bottle", "No night-night" and "More cookie".<br>(Note: The examples should be adapted for local relevance.)
LAN19	Does your child say at least six words?	The child is able to spontaneously and appropriately say at least six meaningful words that apply to a specific object or situation.

Item code	Item question	Description
		(Note: If the caregiver is unsure, you can ask her to list the words the child knows. A word is considered meaningful if it is consistently used and understood for a specific object in the home.)
LAN20	Does your child understand the words over/ on top of/ above, next to, under and in front of?<br><br>*If you ask your child to put something under the table or on top of the chair, does he/she understand?*	The child is able to understand if you ask him/her to put something under the table, on top of the chair, next to the cup, behind the cup, or in front of the cup. The child is able to understand at least two of these expressions.
LAN21	When looking at pictures, if you say to your child 'what is this?', does he/she SAY the name of the object that you point to?	The child is able to name at least one picture of an object while looking at a book or a calendar. The word does not have to be said completely correctly as long as it is unique for the object (e.g., "moo" for cow).
LAN22*	Does your child identify at least seven objects? For example, when you ask "where is the ball/spoon/cup/cloth/door/plate/bucket etc." does your child look at or point to (or even name) the objects?	The child is able to identify by looking or pointing at least seven objects commonly found around the house (e.g., cup, spoon, plate, pencil or pen, book, ball, soap, torch, candle, etc., to be determined).<br>(Note: If the caregiver is unsure, you can ask her to list the objects the child identifies. The examples of the words should be adapted for local relevance.)
LAN23*	Does your child usually communicate with words what he/she wants in a way that is understandable to others?	The child is able to communicate with at least one two-word utterance his/her own needs or what he/she wants in a way that is understandable to adults and peers. Words that the child may use include "Give me", "Mama baba (bottle) (or food or cup or water)", "Go now", etc.<br>(Note: The examples of the words should be adapted for local relevance.)
LAN24	Does your child ask questions using the words what, which, where, why, or who?	The child is able to correctly use words what, which, where, why, or who without mixing them up.
LAN25*	Does your child speak in sentences that are 3–4 words long?	The child is able to speak in sentences that contain at least three words. Common examples include "Daddy go home", "Give me toy", "Do it again", "I want that" and "Me get ball".<br>(Note: The examples of the words should be adapted for local relevance.)
LAN26	Does your child know the difference between the words "big" and "small"? For example, if you ask, "Give me the big spoon" can your child understand which one to give if there are two different sizes?	The child is able to recognize the difference between the words "big" and "small".

Item code	Item question	Description
LAN27	Does your child talk about or explain things that have happened in the past using the past tense? For example, "I went to the market yesterday" (NOT "I go to the market yesterday").<br><br>*Does your child tell you about something that happened on a last special event? Does he/she talk about anything that happened in the past?*	The child is able to talk about or explain about something that happened in the past (e.g., what the child did yesterday, something that happened at a recent special event). He/she is able to use one or more words to communicate the past tense.
LAN28	Does your child count up to 5?	The child is able to count up to 5 by memory.
LAN29	Does your child understand the term "longest"? For example if you ask him/her to choose "which is the longest of 3 objects?" e.g. 3 spoons or sticks – would he/she be able to choose the longest?	The child is able to differentiate between sizes.
LAN30	Does your child tell a story?	The child can retell a story heard before, using appropriate words to make it recognizable; or can talk about events that happened to him her in a way understandable to others. The story or the event are longer than one sentence and include a beginning and an end with consequentiality.
**SOCIO-EMOTIONAL**		
SE1	Does your child stop crying or calm down when you come to the room after being out of sight, or when you pick him/her up?	The child is able to stop crying or calm down when the caregiver comes to the room after being out of sight, or when he/she picks him/her up.
SE2	When you are about to pick up your child, does he/she act happy or excited?	When you are about to pick up your child, the child is able to act happy or excited (e.g., by smiling, making noises or moving arms or legs).
SE3	If you play a game with your child, does he/she respond with interest?<br><br>*For example if you play peek-a-boo, pat-a-cake, wave bye-bye etc. does your child smile, widen their eyes, kick or move arms or vocalize.*	The child is able to respond with interest to the caregiver playing a game with him/her (e.g., the child smiles, widens his/her eyes, kicks or moves arms or vocalizes when playing peek-a-boo, pat-a-cake, waving bye-bye, etc.). The child maintains attention and enjoys interactions for at least 60 seconds. He/she does not have to participate and carry out the activities but must just show interest.
SE4	Does your child smile when you smile or talk with him/her?	The child is able to smile when the caregiver talks to him/her.
SE5	Does your child smile, move excitedly, kick legs, move arms or trunk, or make coo noises when a known person enters the room or speaks to him/her?	The child is able to smile, move excitedly, kick legs, move arms or trunk, or make coo noises when a familiar person enters the room or speaks to them.
SE6	Is your child interested when he/she sees other children playing?<br><br>*Does he/she watch, smile, or look excited?*	The child is able to show interest when seeing other children playing, by following them with his/her eyes, smiling, making gestures in response to seeing others doing so.

Item code	Item question	Description
SE7	Is your child very interested to play with a new toy or object?	The child is able to show excitement (e.g., by moving, smiling, shaking, vocalizing, reaching for toy, etc.) when there is a new toy or object. Score YES if child shows some excitement.
SE8	Does your child usually get along with other children he/she plays with such as siblings or other family members?<br><br>*When your child spends time with other children, does he/she mostly agree on things and not quarrel?*	The child is able to get along well with other children most of the time and to make friends easily. When he/she spends time with other children, they mostly agree on things and do not quarrel.
SE9	When your child sees other children playing, does your child play next to them without joining in?<br><br>*Does he/she play alongside?*	The child is able to play next to other children playing, even if he/she does not join in the game. He/she plays alongside.
SE10	Does your child play together with other children not just by sitting next to each other and doing the same thing, but actively interacting?	The child is able to play together with other children in ways that involve direct interactions and engagement.
SE11	When your child needs to use the toilet, does he/she show you by pulling on his/her clothes, holding him/herself, crying, or some other way?	The child is able to show to the caregiver that he/she needs to use the toilet by using gesture (e.g., pulls pants, etc.) or tells caregiver in some way that they must use the toilet. If a child is verbal, using language counts too.
SE12	Does your child share things with other children or family members without being told to do so?	The child is able to share objects he/she cares about with other children or family members without being told to do so.
SE13	Does your child show sympathy or look concerned?<br><br>*If your child's friend or sibling is sad, what does your child do? Does your child try to comfort the other?*	When someone familiar gets hurt or is sad within the child's sight, the child is able to show sympathy or look concerned. For example, your child may look visibly upset if a friend starts crying, or may go to the friend in an effort to comfort the other. The child looks upset in reaction to someone else being sad or hurt but does not necessarily have the capacity to express and communicate why they are upset.
SE14	If your child has a problem with another child, fighting over a toy, or something else, does your child come to you or another adult and ask for help?	The child is able to ask adults for help for something that he/she is not able to do, or for resolving a conflict.<br>(Note: The child is looking to solve a problem and/or resolve a conflict. It measures help seeking; simply complaining about someone else does not count for this item.)
SE15*	Does the child usually show respect for authority figures (e.g., teacher, grandparent, elder)?	The child is able to control his/her impulses and follow instructions given by an authority figure.<br>(Note: The examples of the authority figures should be adapted for local relevance.)

Item code	Item question	Description
SE16	Does your child worry if a known person is sick or hurt?<br><br>*Is child interested in the wellbeing/health of others?*	The child appears upset if someone they know is ill or hurt. For example, child asks what happened to someone that got sick and is not there or is in bed, and the child shows signs of concern for whether they will get better. The child seems to understand that something is wrong with the person and wants to know more about it. He/she expresses concern visibly or verbally.<br>(Note: The reaction of the child is more nuanced than SE13.)
SE17*	Does your child follow through on instructions, for example, does he/she finish his/her chores?	If given an instruction or simple task that can be done by a child that age (for example, putting objects into a basket), the child completes it.<br>(Note: The examples of the chores should be adapted for local relevance.)
SE18	Does your child keep working on something until he/she is finished?	The child is able to complete tasks that he/she initiated without getting distracted.
SE19	Does your child use "imaginary" or not visible objects in play?<br><br>*For example, pretending to talk on a cell phone or pretending to give food to a child (even though they have nothing to give)*	The child is able to use imaginary objects in his/her play. Examples include: Putting an imaginary bandage on a teddy bear; brushing a doll's hair with an imaginary comb; cupping one hand as a bowl and using an imaginary spoon to eat imaginary soup or porridge. He/she displays a novel or spontaneous imaginary play, which is different from only representational play (e.g., uses blocks to represent food).
SE20	Does your child have difficulty taking turns when playing with others?	The child may get agitated if his/her needs or wants are not immediately met; child tries to push in front of other children; or always wants to do things first.
**GENERAL BEHAVIOR**		
GB1	Does your child pay attention when someone is talking to him/her?<br><br>*Does he/she look and respond to you when you are talking or asking child to do something?*	The child is able to look at a person interacting and responds by looking, smiling, cooing, moving body, etc.
GB2	Does your child sit or play quietly on his/her own for at least several minutes?	The child is able to entertain him/herself for several minutes (at least 5) without any electronic device or demanding assistance from others.
GB3	Does your child hold on to your and has a hard time leaving your side?	The child is excessively clingy to the caregiver, has a hard time being away from the caregiver and is uncomfortable when out of the caregiver's direct presence, even if in a familiar setting. He/she holds on to the caregiver and does not want him/her to

Item code	Item question	Description
		leave. The child constantly needs physical closeness or becomes upset when caregiver is away, even if just for a few seconds (at least 10).
GB4	Does your child get upset when the caregiver leaves the room?	The child gets very upset when a caregiver leaves the child's sight, even if others of any age are still in the room. He/she protests strongly, crying and fussing and has a hard time settling when the caregiver tries to comfort him/her upon return.
GB5	Does your child get upset when the caregiver leaves him/her with someone else?	The child gets upset when caregiver makes an explicit attempt to leave them with another person not necessarily known to the child. He/she protests strongly, crying and fussing, and has a hard time settling when the caregiver tries to comfort him/her upon return. (Note: Behaviors are similar as in GB4, but the context is different.)
GB6	Does your child take an object and pretend it is something else?<br><br>*For example, does your child use something to pretend it is a phone?*	The child is able to pretend that an object is something else (e.g., he/she uses something to pretend it is a phone, or a doll, or knife, or food, or blanket, or broom; cloth used as a blanket, pillow, or diaper; block used as salt or pepper dispenser; cup used as a drum or a hat).
GB7	When a known adult asks your child to do something, does he/she comply?	The child is able to comply with a demand made by a familiar adult (e.g., when asked to bring something, greet someone, get something from another room or area).
GB8	Does your child ever try to imitate your actions around the house?	The child is able to try to pretend to do actions they see adults do: sweeping, cooking, or washing, driving, talking on the telephone.<br>(Examples to be provided locally to ensure relevance and gender equity.)
GB9	Does your child play well in a group of children?	The child is able to play together with more than one child at a time without getting into conflicts immediately.
GB10	Does your child kick, bite, or hit other children or adults?	The child is often aggressive toward other children or adults. He/she kicks, bites, hits, etc.
